# A High-Throughput Search for Composition–Phase–Property Relations of Cu–Ni/Ti–Al Elastic Copper Alloys

**DOI:** 10.3390/ma11122513

**Published:** 2018-12-11

**Authors:** Wang Jianwei, Xiao Wei, Zhang Chuan, Sun Lu, Huang Guojie, Shi Jingmin, Wang Ligen

**Affiliations:** 1 Materials Computation Center, GRIMAT Engineering Institute Co., Ltd., Beijing 101407, China; wxiao@ustb.edu.cn (X.W.); sunluicey@126.com (S.L.); shijm03@163.com (S.J.); 2 Compu Therm, LLC, 437 S. Yellowstone Dr., Madison, WI 53719, USA; chuan.zhang@computherm.com; 3 State Key Laboratory of Nonferrous Metals and Processes, GRIMAT Engineering Institute Co., Ltd., Beijing 101407, China; huangguojie@grinm.com; 4 GRIPM Advanced Materials Co., Ltd., Beijing 101407, China; lg_wang1@yahoo.com

**Keywords:** elastic copper alloy, high-throughput, diffusion multiple, CALPHAD

## Abstract

A high-throughput method was employed to effectively obtain the cross-scale relationship of elastic copper alloys. Firstly, a Cu–Ni–Ti–Cu25Al–Cu35Sn diffusion multiple was prepared and heat-treated under a specified condition to form a series of diffusion layers with the concentration gradient at the multielement metal interface. Then, the compositions, elastic moduli, and hardness of the Cu–Ni–Al and Cu–Ti–Al ternary copper alloys were tested. Meanwhile, the solid phase sequences in the diffusion zones were predicted by the CALPHAD (CALculation of PHAse Diagram) method. Through these experimental and calculated results, the composition–phase–property relations of the Cu–Ni–Al and Cu–Ti–Al ternary systems were established.

## 1. Introduction

The beryllium copper elastic alloy has excellent mechanical properties [1,2,3] and is also well known for its good conductivity, heat conduction, impact resistance, and magnetic field resistance. It is widely used in precision equipment in the aerospace, electronics, and power transmission industries, as well as others [4]. However, beryllium and its oxides are toxic, which can cause human acute or chronic beryllium poisoning. Obviously, beryllium copper alloys, the beryllium content of which is 0.4–2.8 wt.%, pose huge toxic hazards in the preparation, application, and recycling processes. Additionally, the production cost of beryllium copper alloy is high. Therefore, it is necessary to develop new environmentally friendly elastic copper alloys to replace beryllium copper alloys.

Since the 1950s and 60s, Cu–Ti–M and Cu–Ni–M alloys have been widely studied [5,6,7]. Among them, the Cu–Ti alloy [8,9] shows excellent performance and, particularly, a high conductivity. The Cu–Ti alloy is often used to make parts with high strength, high elasticity, and high wear resistance. Cu–Ni–Ti, which is a precipitation strengthening alloy similar to the beryllium copper alloy, has the potential to be developed as a new elastic material. The Cu–Ni–Sn alloy also has a wide scope of application because of its excellent properties and medium-level strengths [10,11,12], but the segregation of Sn occurs easily and the preparation process is complicated.

Although much research has been done on these elastic copper alloys, the results have been unsystematic and incomplete. Also, the relationship between the phase composition and elastic properties of these alloys is not thoroughly understood. In addition, many potential alloy varieties are not yet known and have not been investigated. This is mainly due to the limitations of conventional experimental techniques [13,14]. Compared to the conventional one-alloy-at-a-time approach, combinatorial approaches dramatically increase the efficiency of materials discovery by coupling parallel syntheses of large libraries of compositions with effective screening for desired properties, such as elasticity and hardness [15,16,17,18,19,20,21].

In this work, the diffusion multiple was employed to create large libraries of compositions in the bulk sample for fast and systematic studies of Cu–Ni–M and Cu–Ti–M properties. Specifically, we tried to establish the composition–phase–property relations of Cu–Ni–Al and Cu–Ti–Al elastic copper alloys by a high-throughput search with microanalytical techniques.

## 2. Experimental Approach

**Figure 1:**
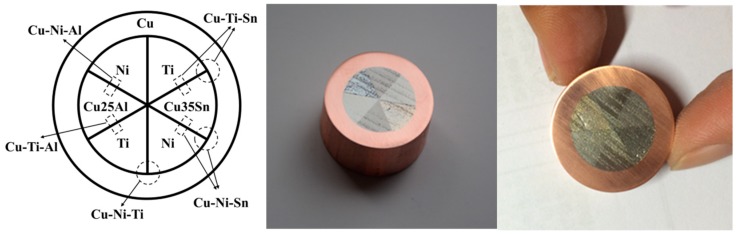
Cross section of Cu–Ni–Ti–Cu20Al–Cu35Sn diffusion multiple.

The starting pure elements were melted with a purity of 99.99 wt.% for Cu, 99.98 wt.% for Ni, 99.995 wt.% for Ti, 99.99 wt.% for Al, and 99.99 wt.% for Sn. Among them, the melting temperatures of Sn and Al are 231 and 660 °C, respectively, both of which are lower than those of Cu and Ni. To avoid the liquid phase occurring during the diffusing heat treatment, the Cu-35 wt.%Sn (Cu35Sn) and Cu-25 wt.%Al (Cu25Al) alloys were used in place of pure Sn and Al. The metals and alloys were processed into a specified shape, and pure copper was used as a package. All oxide layers on the component surfaces were removed through surface treatment. The components were assembled [10] after demagnetization. The cross interface of the diffusion multiple is shown in Figure 1. Several ternary systems, including Cu–Ni–Al, Cu–Ti–Al, Cu–Ni–Ti, Cu–Ni–Sn, and Cu–Ti–Sn, were concurrently investigated from this diffusion multiple. Here, the Cu–Ni–Al and Cu–Ti–Al systems are discussed to illustrate the experimental approach.

The sample was prepared by a conventional way proposed by Zhao [19], firstly being packaged by vacuum electron beam welding, and then being subjected to 4 h of hot isostatic pressing at 700 °C and 200 MPa for metallurgical bonding. The specimen was mechanically cut along the cross section into a number of flaky specimens, which were then vacuum encapsulated in a quartz tube. The packaged sample was heat-treated for 400 h at 650 °C. After that, the morphology of the metal interfaces was observed by a Carl Zeiss EVO 18 high-resolution scanning electron microscope (SEM, Carl Zeiss Microscopy GmbH, Oberkochen, Germany). The acceleration voltage of the scanning electron microscope was 20 KV. The HYSITRON TI-900 nanoindentation (Hysitron Inc., Minneapolis, MN, USA) was used to make a series of indentations and to obtain the in situ hardness and elastic modulus data. The spacing between the indentation points was 5 μm, and the depth of each indentation was 0.5 μm. The element distribution in the diffusion zone was scanned by a JXA-8230 EPMA (Electron Probe Micro-analyzer) electron probe instrument (JEOL, Tokyo, Japan). The acceleration voltage was 0.2–30 KV and the probe current was 10^−12^ to 10^−5^ A.

Meanwhile, the CALPHAD approach [22,23,24,25,26] was used to predict the solid phase sequences in the diffusion zone of the multiple. The thermodynamic calculations were carried out using PANDAT software [27,28] and related commercial databases.

## 3. Results and Discussion

As shown in Figure 1, six blocks in the form of sectors of a cylinder were welded together under pressure, and multiple constituents were found in the interdiffusion zone near the point where the sectors met. The high-throughput measurements, which provided data of high sensitivity and spatial resolution, could determine the hardness and elastic moduli as the concentration increased. By the comparisons with the thermodynamic calculation results, the relationships of the phase, the composition, and the properties of the copper alloys were examined in a high-throughput way.

### 3.1. Cu–Ni–Al System

Here, the Cu25Al–Ni binary diffusion layer in the diffusion multiple was employed to characterize the Cu–Ni–Al ternary.

**Figure 2:**
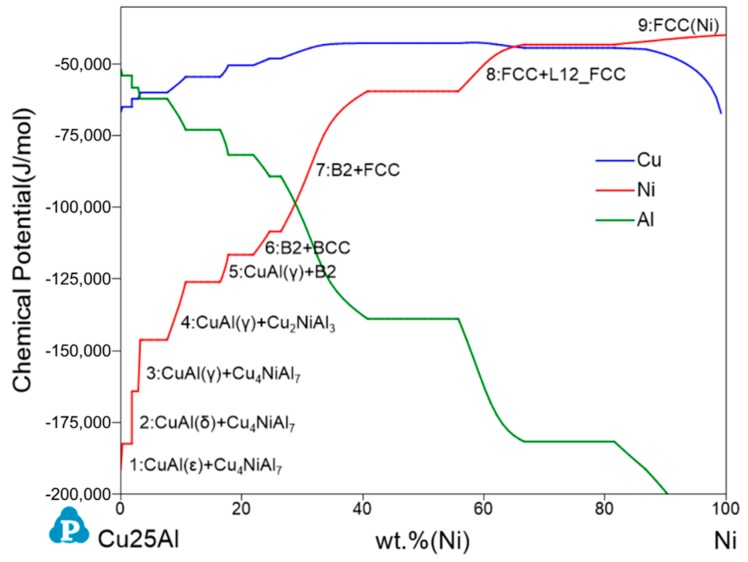
Chemical potentials of Cu, Ni, and Al in the Cu25Al–Ni pseudo-binary at 650 °C.

In order to better understand the Cu25Al–Ni binary diffusion, the thermodynamic chemical potentials of Cu, Ni, and Al as a function of wt.%Ni (Figure 2) at equilibrium were calculated. The plot indicates that Al and Ni were the controlling elements during this diffusion process. It is well known that diffusion is critically driven by a gradient in the chemical potential of the diffusing species, on the basis of which a theoretical solid phase sequence(s) formed in the Cu25Al–Ni binary diffusion zone could be predicted (Figure 2). The curve platform represents three phases of equilibrium and indicates that no chemical potential difference drives the formation of these three-phase regions, supported by Gibbs’ phase rule, which at constant temperature and pressure, is given by F = 3-P, where F is the number of degrees of freedom, 3 is the number of components, and P is the number of phases in thermodynamic equilibrium with each other. Therefore, each interval of the Cu25Al–Ni binary diffusion zone was composed of a single phase or two phases. Further, the calculation results propose a nine-region diffusion zone in theory (Figure 2). However, the real kinetic processing/diffusion path in most cases will not locate exactly on the calculated isopleth mainly because local equilibrium is practically hard to attain at interfaces in limited conditions.

**Figure 3:**
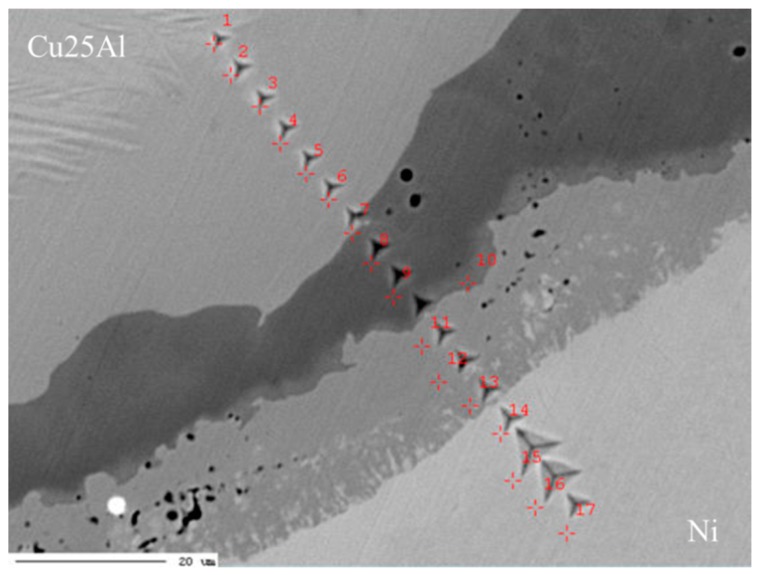
The nanoindentations of the Cu25Al–Ni interface diffusion zone, 650 °C × 400 h.

**Figure 4:**
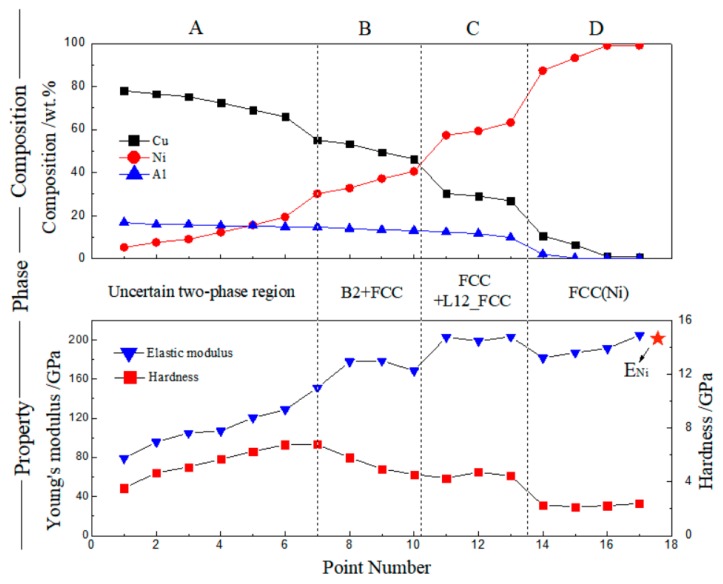
Composition–phase–property relation of the Cu–Ni–Al ternary system, 650 °C × 400 h.

Figure 3 shows the surface morphology of the heat-treated Cu25Al–Ni interface sample with nanoindentations. The indentations are shown along a line that passes through all four main diffusion regions. We combined the composition distribution together with the EPMA test data in Figure 4 and divided the map into four parts (A–D) with different phase compositions according to the indentations in various morphology regions. Therefore, a composition–phase–property relation of the Cu–Ni–Al ternary system can be established following Figure 4. 

Based on the thermodynamic prediction, it is inferred that the phase composition of regions B–D in Figure 4 are B2 + FCC, FCC + L12_FCC, and FCC(Ni) [29,30], respectively. This is because the Ni content of these three regions (Figure 2 and Figure 4) agree well. It should be noted in particular that the calculation represents a large chemical potential range for all these phases or phase groups, which seems to be explained by the chemical potential difference, although lacking theoretical illustrations. Region A is a little complex. The surface morphology reveals a unique phase region, and the property diagram also shows an obvious increasing trend in this part. However, the compositions of the components in region A show an uncertain situation, while the corresponding calculation curve below approximately 40 wt.%Ni shows several possible two-intermetallic groups. It is difficult to determine the detailed phase composition of region A from the above analysis, possibly because of the diffusion limitation in this experimental condition. At present, we roughly assign it as a two-phase region with uncertain phases, which needs to be further determined.

The composition–phase–property relation of the Cu–Ni–Al ternary system is given by Figure 4. On the whole, the elastic modulus and hardness values were consistent with the change trend of Ni and Cu content, in a positive or reverse way. Generally, when two metals with same lattice type and similar valence electrons and atomic radii, such as Cu and Ni, constitute a continuous solid solution, the relationship between the elastic modulus and solute concentration is linear or approximately linear. The last elastic modulus data in Figure 4 are slightly bigger than those of pure Ni (200 GPa) [31] due to the solid solution of a small amount of Cu in Ni. 

### 3.2. Cu–Ti–Al System

**Figure 5:**
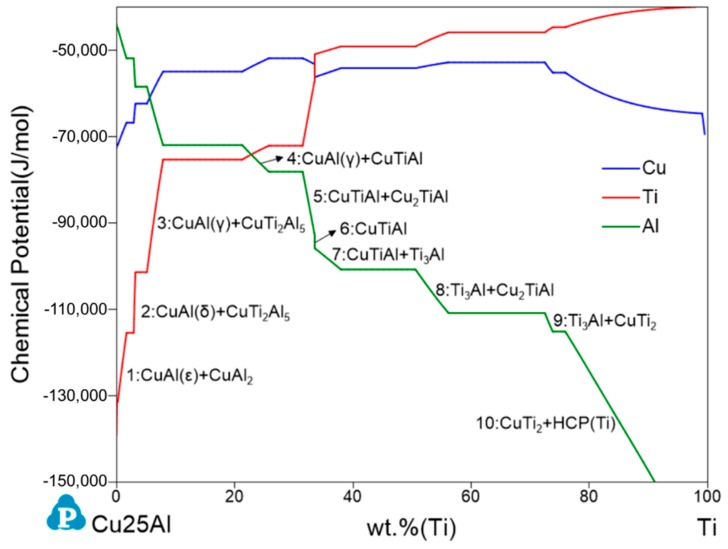
Chemical potentials of Cu, Ti, and Al in the Cu25Al–Ti pseudo-binary at 650 °C.

The Cu25Al–Ti binary diffusion layer in the diffusion multiple was employed to characterize the Cu–Ti–Al ternary. Similarly, the chemical potentials of Cu, Ti, and Al as a function of wt.%Ti at 650 °C were calculated (Figure 5). The diffusions of Ti and Al were the controlling factor during the diffusion process and a 10-region diffusion zone is proposed by Figure 5. 

**Figure 6:**
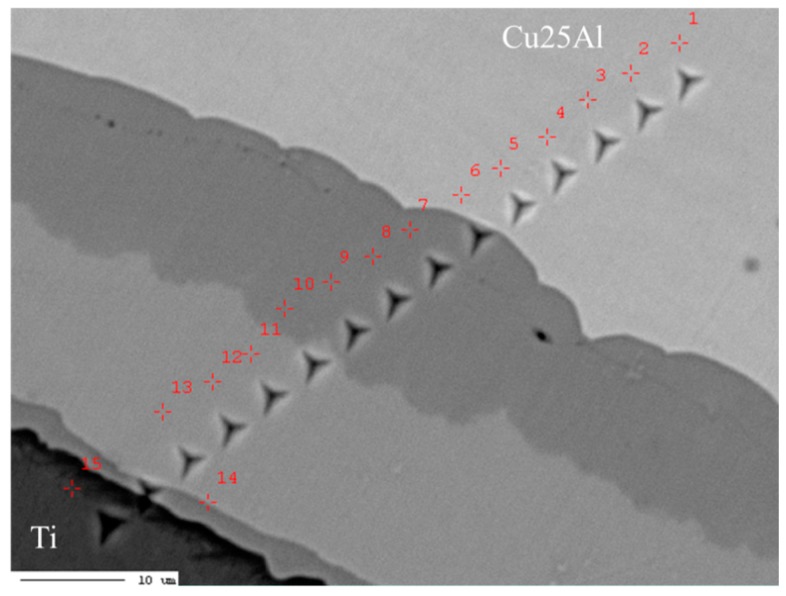
The nanoindentations of the Cu25Al–Ti interface diffusion zone, 650 °C × 400 h.

**Figure 7:**
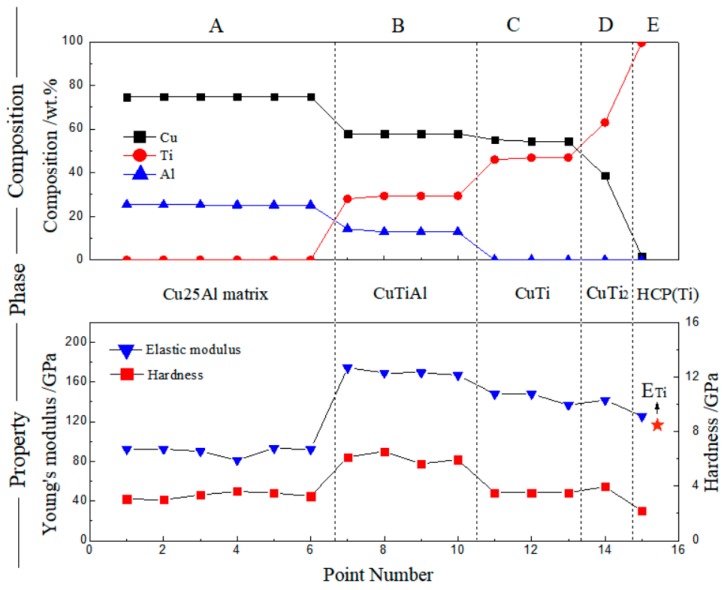
Composition–phase–property relation of the Cu–Ti–Al ternary system, 650 °C × 400 h.

Figure 6 shows the surface morphology of the heat-treated Cu25Al–Ni interface sample with nanoindentations. Five parts can be recognized by the morphology difference. Therefore, the composition map in Figure 7 is divided into regions A–E.

It is obvious that region A and E present the matrix Cu25Al and pure Ti, respectively. Regions B–D are difficult to identify. Region B is the only ternary region in Figure 7. According to the calculation, the phase composition of region B should be CuTiAl [32], while regions C and D suddenly turn into totally binary systems, as shown in Figure 7. They are different from the theoretical solid phase sequence(s) given by Figure 5, which show a gradual process of phase transformation. This is probably because the diffusion of Al was not sufficient during the 400-h annealing treatment. It is also believed that this may be due to the heat-treatment conditions described in this paper. When the interface nearly reached a local equilibrium, the CuTiAl phase had the largest driving force and preferentially precipitated. Therefore, region B was followed by the Cu–Ti binary system that practically did not include any Al components. On this basis, regions C and D tended to be composed of CuTi and CuTi_2_ [32], respectively, according to the content ratio of Cu/Ti. The modulus and hardness data give similar divisions from the property panel of Figure 7. The composition–phase–property relation of Cu–Ti–Al ternary is proved by this agreement. 

Similar to the case of the Cu–Ni–Al system, the last elastic modulus (Figure 7), corresponding to the HCP(Ti) phase with a small amount of Cu solution, was a little bigger than the modulus of pure Ti (116 GPa). Additionally, the modulus and hardness of the Cu–Ti–Al system varied in a similar trend. In contrast, for Cu–Ni–Al (Figure 4), they varied in an opposite way on the whole. Generally, the determinant factor of the hardness is the structure, and it is the interatomic binding force that determines the modulus. Therefore, the different trend in these two systems can be explained.

The Cu–Ni–Ti copper alloy, as a widely accepted candidate, is not discussed here. Meanwhile, because the copper alloy containing Sn has its particularities regarding segregation and strengthening, the Cu–Ni–Sn and Cu–Ti–Sn ternaries are also not discussed here, and they will be the subject of our future investigations.

## 4. Conclusions

To efficiently obtain the composition–phase–property relationship of several copper alloys, a Cu–Ni–Ti–Cu25Al–Cu35Sn diffusion multiple was made. Through solid-phase diffusion, a series of diffusion layers with a concentration gradient were formed at the multielement metal interface. Using the nanoindentation and electron probe testing method in the diffusion zone, the compositions, elastic moduli, and hardness of the Cu–Ni–Al and Cu–Ti–Al ternary copper alloys were examined in a high-throughput way. The CALPHAD method was used to predict the solid phase sequence(s) thermodynamically. On the basis of these results, a series of reasonable composition–phase–property relations of Cu–Ni–Al and Cu–Ti–Al ternary systems was established. The method used in this work could be widely applied in other multicomponent alloys to effectively obtain the cross-scale relationship of the materials.
